# The Influence of Prior Perception, Attitude, and Immediate Knowledge of AI on Adolescents’ Preferences for High- and Low-Replaceable Jobs

**DOI:** 10.3390/bs16010072

**Published:** 2026-01-05

**Authors:** Huanlei Wang, Xiaoxiong Lai, Shunsen Huang, Xinran Dai, Xinmei Zhao, Yun Wang

**Affiliations:** 1 State Key Laboratory of Cognitive Neuroscience and Learning, Beijing Normal University, Beijing 100875, China; wanghuanlei@mail.bnu.edu.cn (H.W.); daixinran@mail.bnu.edu.cn (X.D.); zhaoxm@mail.bnu.edu.cn (X.Z.); 2 Institute of Digital Education, China National Academy of Educational Sciences, Beijing 100088, China; laixx@cnaes.edu.cn; 3 Institute of Sociology, Chinese Academy of Social Sciences, Beijing 100732, China; huangss@mail.bnu.edu.cn; 4 Chengdu Shisunjie Primary School, Chengdu 610031, China; 5 Guangdong Experimental High School, Guangzhou 510000, China

**Keywords:** Artificial Intelligence, familiarity with AI, trust in AI, attitude towards AI, adolescents, career preference

## Abstract

While extensive research has examined adult perceptions of Artificial Intelligence (AI)’s impact on the workforce, studies focusing on adolescents—who are at a critical stage of career development—remain scarce. Drawing upon Social Cognitive Career Theory (SCCT) and the significance of fostering adolescents’ adaptability in the era of AI, we designed a questionnaire-based experiment to examine how adolescents’ prior perceptions, attitude and immediate knowledge of AI influence their career preferences. We conducted a questionnaire-based experiment with 836 adolescents (*M*_age_ = 13.98, *SD* = 1.35 years; 52.30% male) to investigate the influence of three independent variable groups: prior perception of AI (familiarity with AI), prior attitudes towards AI (trust in AI and positive/negative attitude towards AI) and immediate knowledge of AI (experimental manipulation). The results showed that immediate knowledge of AI significantly and negatively predicted adolescents’ preference for both low- and high-replaceable jobs. In contrast, familiarity with AI, trust in AI and positive attitude towards AI significantly and positively predicted a preference for low-replaceable jobs. This study introduces an integrated empirical framework demonstrating that distinct AI-related factors differentially influence adolescent career preferences. Results emphasize the importance of familiarity with AI, trust in AI and positive attitude towards AI among teenagers in order to better adapt the future changes in the labor market. This finding provides an empirical reference for educators and policy makers to better guide teenagers to make career plans.

## 1. Introduction

Artificial Intelligence (AI) is a broad field, and some researchers defined AI from a technical perspective as a powerful and complex technology designed to simulate human intelligence (Glikson & Woolley, 2020). Some researchers defined AI as the simulation of human intelligence processes by machines, including learning, reasoning, and self-correction (Gillath et al., 2021). We adopted the definition of AI used in related research (Gillath et al., 2021) to define the scope of AI in our study and presented this definition to the participants in the questionnaire: AI is the simulation of human intelligence processes using machines, particularly computer systems. These processes include learning (automatically acquiring and utilizing information), reasoning (drawing approximate or definite conclusions based on rules), and self-correction. AI has a wide range of applications, including expert systems, speech recognition, and machine vision. Specific examples of AI include personal assistants (such as Siri, Xiao Ai, Xiaodu, Tmall Genie, etc.), medical diagnostic aids, self-driving vehicles, and intelligent robots.

AI is becoming increasingly pervasive and has even had a profound impact on human decision-making (Duan et al., 2020), work (Yin et al., 2024) and mental health (Huang et al., 2024). However, the widespread adoption of AI technologies has raised concerns about job displacement, with many people fearing that AI will take their jobs (Fleming, 2019). Especially with the emergence of ChatGPT (GPT-3), there has been a lot of discussion about the impact of AI on the future of work and employment (George et al., 2023). According to a report published by the McKinsey Global Institute, approximately 375 million people will be replaced globally in the future, and as many as 50% of the world’s jobs could be replaced by robots, with 60% of those jobs and 30% of jobs being performed by machines (Manyika et al., 2017). According to the latest report, AI technology has the potential to both replace certain professions and create new forms of work through task augmentation, and the key lies in understanding AI, learning AI, and enhancing one’s abilities to adapt to the changes in the AI-driven labor market (United Nations, 2024).

AI presents both opportunities and risks and has the potential to have a greater impact on many aspects of adolescents (Hasse et al., 2019; JAGW, 2023; UNICEF, 2021). Innovations in AI are helping to reshape the labor market, with important implications for career trajectories, and using AI is a necessary skill for young people to thrive in an ever-changing work environment (Hasse et al., 2019). A recent survey by Junior Achievement of Greater Washington (JAGW, 2023) contextualizes this concern, highlighting that as AI makes strides in tasks central to many occupations, 66% of adolescents are concerned that they will not be able to find a good job when they grow up. Significant career development occurs during adolescence, when individuals begin to define their vocational identity (Erikson, 1964); during adolescence, individuals attempt to assume career roles, engage in career planning and career exploration, and make career decisions (Super, 1980). Adolescence is a critical stage for shaping career trajectories through the development of interests (Hoff et al., 2022). Due to the rapid development of robotics and AI leading to technological unemployment and significant uncertainty, researchers insist on the need for youth-focused coping strategies (Pol & Reveley, 2017). Research also indicates that adolescents are more prone to career indecision due to the abundance of career options and their lack of experience (Priyashantha et al., 2023). Therefore, in the context of the rapid development of AI, it is particularly important to explore the impact of AI on adolescents’ career preference.

Previous studies have explored adolescents’ concerns about their future careers in the AI era (Hasse et al., 2019; JAGW, 2023) and how enhancing their AI literacy and some coping strategies can prepare them for their professional futures (Pol & Reveley, 2017; Zhang et al., 2023). However, current studies have the following two gaps. The first gap concerns the prior factors: there is a lack of overall understanding regarding adolescents’ general perceptions and attitudes toward AI. Current research often focuses on a certain kind of products, and the results are also varied. Therefore, it is difficult to comprehensively evaluate how adolescents’ general perceptions of and attitudes towards AI influence their career preferences. For example, one study shows that 14.8% of adolescents reported using some form of generative AI to complete their schoolwork (Klarin et al., 2024), while a report suggests that previous perceptions and attitudes may vary significantly: not all adolescents are familiar with digital technologies and AI systems, and some have never heard of AI (UNICEF, 2021). Understanding the perception and attitude of AI on their career preferences can help us better predict and let them prepare well for careers in the AI era. The second gap concerns the immediate factors: the impact of immediate knowledge (the dissemination of immediate knowledge in the AI environment) on individuals remains an important issue to be discussed. For example, existing studies suggest that fear developed through the media can be a barrier to learning and accepting AI (Liang & Lee, 2017). So do adolescents experience either overconfidence or panic towards career preference in the AI era due to a lack of AI knowledge? But there is limited research on whether this kind of immediately gained knowledge of AI can change their career preferences. Therefore, this study uses questionnaires and experimental methods to address these two gaps. We examine how prior perception of AI (familiarity with AI), prior attitudes towards AI (trust in AI, positive and negative attitudes towards AI) and the provision of immediate knowledge of AI (experiment manipulation) influence adolescents’ career preferences.

## 2. Theoretical Background and Hypotheses

### 2.1. Person–Environment (P–E) Fit Theory and Social Cognitive Career Theory (SCCT)

Grounded in Person–Environment (P–E) Fit Theory (Hesketh & Gardner, 1993), individuals are posited to achieve better adjustment and satisfaction when their personal characteristics (e.g., abilities, values) align with the characteristics of their work environment (e.g., demands, supplies). First, regarding prior perceptions and attitudes, adolescents with greater familiarity and more positive attitudes toward AI may perceive a stronger fit between their own cognitive preparation and the requirements of careers that heavily utilize or collaborate with AI. Kong et al. (2023) indicates that employees who trust in AI tend to exhibit positive behaviors in collaboration with it. This trust not only enhances their job satisfaction but also helps them better adapt to environments where AI is prevalent. Secondly, regarding immediate knowledge of AI, the acquisition of new AI knowledge may lead adolescents to reassess their career environment by influencing their perceived alignment between personal characteristics and the demands or rewards of the environment. Therefore, we want to examine whether immediate AI knowledge influences adolescents’ career preferences.

Social Cognitive Career Theory (SCCT) indicates that individuals’ past learning experiences influence their self-efficacy and outcome expectations, thereby affecting career interests and goal setting, with contextual factors moderating these processes (Lent et al., 1994; Lent & Brown, 2006). According to SCCT, adolescents’ prior perceptions of and attitudes toward AI influence their self-efficacy and outcome expectations, thereby affecting their willingness to choose AI-related careers. Additionally, SCCT highlights the role of contextual affordances and barriers. The dynamic environmental factors in SCCT (Brown & Lent, 2017) suggest that immediate knowledge about AI as a contextual factor impacts adolescents’ outcome expectations, prompting them to adjust their interest in different careers based on new information, ultimately leading to more adaptable career preferences.

### 2.2. Prior Perception of AI and Attitudes Towards AI Influence Career Preference

Building on Social Cognitive Career Theory (Lent et al., 1994), we posit that familiarity with AI—as an accumulation of prior learning experiences—shapes career-related cognitions, which in turn influence career preferences.

Familiarity is a person’s understanding of an entity, and it is based on prior experience (Gefen et al., 2003). First, familiarity directly shapes core perceptions. Recent evidence indicates that familiarity with AI enhances perceived trust in the technology, which in turn acts as a critical mediator for increasing the intention to use it (Topsakal, 2025). Building upon this cognitive foundation, familiarity further influences evaluative attitudes (Ho et al., 2023). It not only improves the perceived usefulness and ease of use of AI, but also fosters a more positive overall attitude toward its integration (Kučinskas, 2024). In addition, research suggests that familiarity can promote more positive and engaged interactions between children and AI systems, highlighting its role in shaping early experiential learning (Chen et al., 2023). In many workplaces, human–AI teams are increasingly collaborating to improve joint performance and accomplish tasks that they could not do alone (Wilson & Daugherty, 2018). Due to the adoption of AI, employee engagement has decreased (Kong et al., 2021). Research on AI identity threat in the workplace suggests that loss of status, job change, and AI identity are significant predictors of AI being a threat to employees with AI experience in the workplace (Yam et al., 2022). One study found that employees who are aware of or interact with robots in their workplace, either through direct physical experiences or through their awareness of robots’ increasing presence, report higher levels of job insecurity (Verma & Singh, 2022). This suggests that an individual’s perception of AI and robotics, even in the absence of direct physical interaction, can shape their attitudes towards career stability and may influence their career preferences. Therefore, familiarity with AI not only influences people’s attitudes and acceptance of AI but also affects their career preferences by shaping their perceptions of job security and career prospects.

We hypothesize that adolescents’ attitudes toward AI (encompassing both positive and negative evaluations) significantly influence their career preferences. AI is developing its capabilities at an increasing rate, but it is also creating opportunities at an increasing rate, and research shows that exposure to robots making people feel anxious about job insecurity may be largely due to subjective assessments (Yam et al., 2022). This can be supported by a study: some people feel that machines could threaten their jobs; one study shows that employees do not usually see STARA (smart technology, AI, robotics, and algorithms) as a threat (Brougham & Haar, 2018). Research on technology acceptance indicates that positive attitudes strongly predict an individual’s willingness to use and interact with a technology (Marangunić & Granić, 2015). Therefore, we infer that attitudes towards AI (positive and negative) will influence career preferences.

Drawing on Social Cognitive Career Theory, we posit that trust operates primarily by shaping outcome expectations associated with future work environments. Trust in AI is similar to interpersonal trust, which is influenced by two paths: affective and cognitive. It is the core of human–AI interaction (Jacovi et al., 2021). Trust in AI can predict a positive attitude towards AI and perceived usefulness, which is related to a greater willingness to use it (Choung et al., 2023). However, humans have a low tolerance for AI mistakes, and once an AI makes a mistake, it is difficult to build trust (Dietvorst et al., 2015). Poor AI performance can reduce people’s trust in themselves, which can affect people’s decisions to accept or reject AI advice (Chong et al., 2022). Therefore, we hypothesize that trust in AI can differentially shape positive or negative outcome expectations for different types of work, which in turn influences adolescents’ career preferences.

### 2.3. Immediate Knowledge of AI Influences Career Preference

Researchers proposed a taxonomy of difficulties in career decision-making, including lack of readiness, lack of information, and inconsistent information (Gati et al., 1996); this helps explain that, in uncertain or complex environments (such as the AI era), adolescents may encounter obstacles in career decision-making due to a lack of information or conflicting information, ultimately affecting their career preferences or decisions. As a meta-framework, Systems Theory Framework (STF) provides a holistic perspective on career development, viewing it as a dynamic and environment-dependent process shaped by various influencing systems (individual, social, and environmental) (McMahon & Patton, 2019; Patton & McMahon, 2006); adolescents’ career preferences may evolve and adjust with the input of new information, especially in a rapidly changing AI environment. Apart from this, a study indicates that career preparation can be seen as a multidimensional concept, including career readiness attitudes, knowledge and skills, and behaviors (Steiner et al., 2019), and the study further emphasizes the importance of career knowledge: offering career-related knowledge and training in vocational skills could significantly impact a group of adolescents who largely lack these insights and abilities. But currently, adolescents have different levels of knowledge of AI. A notable proportion of adolescent participants lack familiarity with digital technologies and AI systems, including some who have never even heard of AI (UNICEF, 2021). A Stanford University report reveals a notable gap in access to computer science (CS) education, with students in suburban areas having significantly greater opportunities than their peers in urban and rural districts (Perrault & Clark, 2024). Therefore, we infer that providing AI knowledge to adolescents will influence their career preferences.

### 2.4. The Present Study

This study uses the questionnaires and experimental approach with a large sample of adolescents to investigate whether the prior perception of and attitudes towards AI and immediate knowledge of AI will influence adolescents’ career preference.

**H1:**  *The prior perception and attitudes of AI will influence adolescents’ career preference.*

**H2:**  *Immediate knowledge of AI will influence adolescents’ career preference.*

The independent variables are prior perception of AI (familiarity), prior attitudes towards AI—positive attitude; negative attitude—and trust), and immediate knowledge (AI job replacement crisis). The dependent variable was indexed by willingness scores for high and low-replaceable jobs.

## 3. Methods

### 3.1. Participants

We collected data at a middle school in southwest China. The school has 11 campuses, 160 classes and more than 8500 students. Our study employed a convenience sampling design. After obtaining permission from the school administration to enter the campus to recruit participants, we randomly selected 18 classes from this school and distributed participant recruitment advertisements to all students in these classes. Formal invitations were sent to students who expressed an interest in participating in the study, including themselves and their legal guardians. A total of 879 adolescents participated in this study. To prevent the results from being confounded by careless responses, this study employs the high-precision Mahalanobis distance method to perform outlier analysis on collected multivariate response patterns (Goldammer et al., 2020). The students’ response patterns were analyzed using the open-source *careless* R-package (Yentes & Wilhelm, 2018). This analysis effectively identifies individuals whose response patterns significantly deviate from the majority. Specifically, we established a binary classification rule based on calculated Mahalanobis distance values (using virtual coding: careless responses marked as 1, careful responses marked as 0) to objectively categorize response quality. A total of 43 careless students were excluded. The final number of participants included in the following analyses was 836, and the mean age was 13.98 years (*SD* = 1.35), with an age range from 10.25 to 17.42 years, and 52.30% of the participants were male. All participants’ native language is Chinese, and the ethnicity is the same. The demographic information of the included and excluded participants and the results of the chi-square difference test are shown in Table 1. There was no significant difference between included and excluded participants in terms of gender with a small effect size (Cramer’s V = 0.06), in residence with a small effect size (Cramer’s V = 0.03), in whether they are the only child with a small effect size (Cramer’s V = 0.04).

### 3.2. Procedure

**Figure 1:**
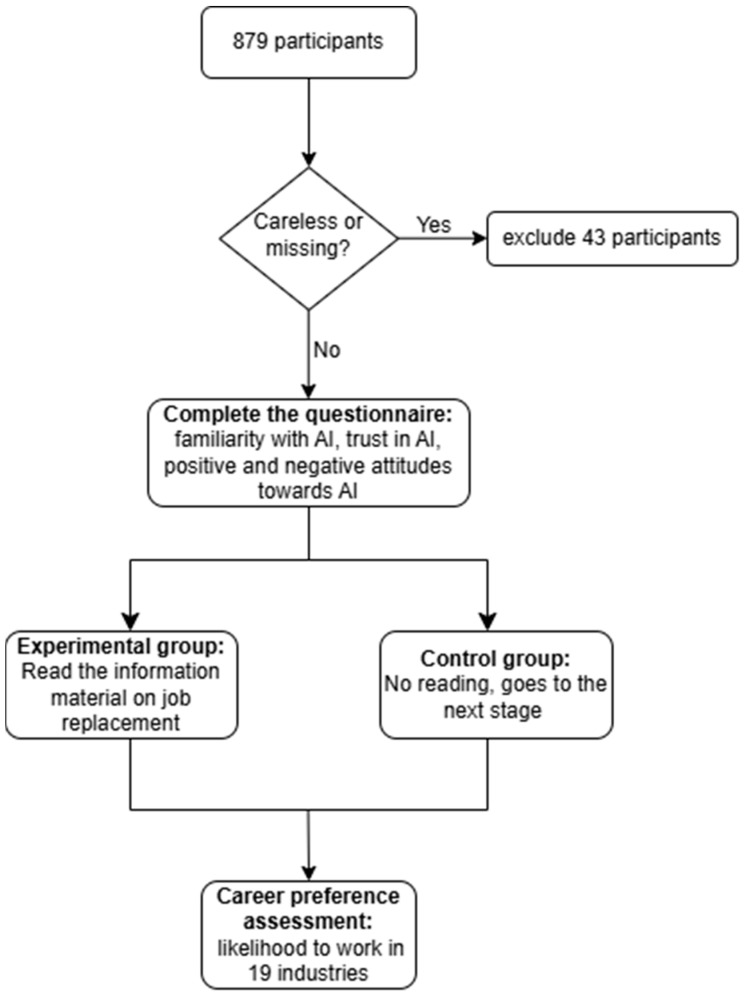
The flowchart of the experiment.

The adolescents completed paper questionnaires independently in the classroom. The experiment was divided into three stages. Stage 1: all participants were asked to complete the questionnaire on the prior perception of AI: familiarity with AI; and the attitude towards AI: trust in AI, and positive and negative attitudes towards AI in turn. Stage 2: after completing the questionnaire, the participants were randomly divided into two groups. This was achieved through a randomized questionnaire distribution method. Specifically, we used two versions of the questionnaire: Version A (Experimental Group) included the text about AI replacing jobs, while Version B (Control Group) did not contain this text. The two versions were otherwise identical. Before the session, the Version A and Version B booklets were shuffled together. The full versions of the questionnaires (Version A and B) employed in this study are provided as Supplementary Materials. The instructor then distributed them randomly to students in the classroom, ensuring that each student had an equal probability of receiving either version. Participants were unaware of the version differences. The participants were given the following instructions: “Please read the following paragraph from an internationally renowned research institute that has conducted a study on the possibility of jobs in various industries being replaced by AI in the future, and the results of the study are as follows.” Materials are provided in Appendix A. The control group did not read the material and proceeded straight to the next stage. Stage 3: The two groups of participants were asked about their career preference: How likely are you to work in the following industries when you are around 30 years old? The industry categories included 19 occupations such as manufacturing, construction, education, etc. The flowchart of the experiment is shown in Figure 1. All procedures performed in this study conformed to the ethical standards of the institutional and national research committee and the Helsinki Declaration of 1964 and its subsequent amendments or comparable ethical standards. This study was approved by the Institutional Review Board (IRB) of the State Key Laboratory of Cognitive Neuroscience and Learning of Beijing Normal University (ethics approval number: CNL_A_0003_003). Written informed consent was obtained from all adolescents, parents, teachers, and school administrators in this study.

### 3.3. Materials

#### 3.3.1. Information Material on Job Replacement

The McKinsey Global Institute report (simply referred to as the McKinsey report) includes an analysis of the extent to which jobs can be replaced by AI in the 19 industry classifications identified by the US Bureau of Labor Statistics (Manyika et al., 2017). We asked two Ph.D. students from the Beijing Normal University to prepare the material; they referred to the industrial classification for national economic activities (2017 edition-2019 revision) developed by the National Bureau of Statistics of China, translated the relevant information from the McKinsey report into Chinese, and made one-to-one correspondence with domestic industries. Finally, we obtained 19 industry classifications, such as manufacturing, construction, education, etc. Then we generated graphical information for the experimental group to read at stage 2. And at stage 3, both the experimental group and the control group would report their willingness to work in these 19 industries (1 = very unwilling, 4 = very willing).

#### 3.3.2. Familiarity with AI

To measure familiarity with AI, we used the familiarity dimension of the Social Service Robot Interaction Trust (SSRIT) scale (Chi et al., 2021). The measure of familiarity with AI consists of four questions, such as “I know a lot about AI” and “I am more familiar with AI than others”. Students were asked to rate how much they agreed or disagreed with the statements on a 4-point Likert scale (1 = strongly disagree, 4 = strongly agree), with higher scores indicating more agreement. Cronbach’s alpha was 0.93, indicating good internal reliability. The confirmatory factor analysis (CFA) was performed using Mplus8.3. A single-factor model was specified based on the familiarity dimension of the SSRIT scale, with all four items loading onto a single latent variable. The CFA model was evaluated using multiple fit indices: Root Mean Square Error of Approximation (RMSEA), Comparative Fit Index (CFI), and Tucker–Lewis Index (TLI). The cut-off criteria for acceptable model fit were RMSEA < 0.08, CFI > 0.90, and TLI > 0.90 (L. Hu & Bentler, 1999). The analysis results indicated that the data fit the model well, with RMSEA = 0.04, CFI = 0.99, and TLI = 0.99.

#### 3.3.3. Trust in AI

The Trust in AI scale which we adapted from one study was used to measure trust in AI (P. Hu et al., 2021). This questionnaire consists of 10 items and includes three dimensions: (1) competence (four items, e.g., “My AI is competent and effective in its interactions with me”), and (2) benevolence (three items, e.g., “I believe that my AI would act in my best interest”) and (3) integrity (three items, e.g., “My AI is truthful in its dealings with me”), showing good internal reliability (α = 0.93). Students were asked to rate how much they agreed or disagreed with the statements on a 4-point scale (1 = strongly disagree, 4 = strongly agree), with higher scores indicating greater trust in AI. We conducted CFA, and a three-factor model was specified based on the adapted Trust in AI scale, with each of the three dimensions (competence, benevolence, and integrity) treated as separate latent variables. Each dimension was represented by its respective items: competence (four items), benevolence (three items), and integrity (three items). As indicated by the results, the present data fit the structure well (RMSEA = 0.05, CFI = 0.99, TLI = 0.99).

#### 3.3.4. Positive and Negative Attitude Towards AI

The questionnaire on perceptions of ICT (information and communication technology) used in the International Computer and Information Literacy Study (ICILS) 2018 (Fraillon et al., 2019) was adopted by us to measure adolescents’ attitudes towards AI. The questionnaire consisted of eight items and included two dimensions: (1) positive attitudes (four items, α = 0.91, e.g., “Advances in AI often improve people’s living conditions”), and (2) negative attitudes (four items, α = 0.68, e.g., “Using AI may be dangerous for people’s health”). Students were asked to rate how much they agreed or disagreed with the statements on a four-point scale (1 = strongly disagree, 4 = strongly agree). A higher score indicated a more positive/negative attitude towards AI. In this study, the Cronbach’s alpha was 0.93. To confirm the structure of the questionnaire, CFA was performed using Mplus 8.3. The analysis specified a two-factor model based on the dimensions of positive and negative attitudes towards AI, with each factor measured by four items. As indicated by CFA, the present data fit the structure well (RMSEA = 0.07, CFI = 0.98, TLI = 0.96).

#### 3.3.5. High/Low-Replaceable Job

**Figure 2:**
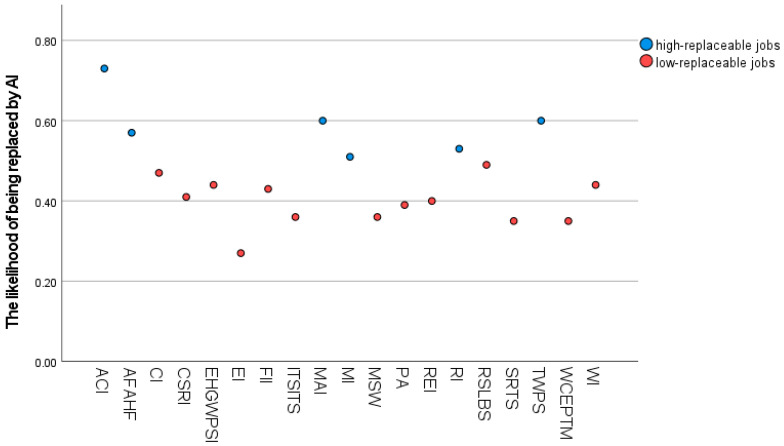
K-means clustering analysis of the likelihood of being replaced by AI across different industries. Blue dots represent high-replaceable jobs (likelihood > 50%), while red dots represent low-replaceable jobs (likelihood ≤ 50%). The X-axis displays the industry codes, and the Y-axis indicates the likelihood score.

To measure participants’ willingness to work in different industries, they were asked: “How willing do you think you would be to work in the following industries when you are about 30 years old?” The industry categories included 19 occupations such as manufacturing, construction, education, etc. Participants were asked to respond on a four-point scale, with 1 being very unwilling and 4 being very willing. For the subsequent analysis, the K-means method was used to cluster the job substitutability, and the industry substitutability score was used as the basis for the cluster analysis, setting the number of categories to two, allowing the statistical software to freely select the initial cluster centers, and reaching the convergence condition after two iterations. Finally, six industries that were ultimately more than 50% replaceable by AI were designated as Category 1 (high-replaceable job in this study), and the rest were designated as Category 2 (low-replaceable jobs in this study), see Figure 2. As we expected there to be variability (as opposed to consistency) within individuals’ responses across these different industries (i.e., adolescents may prefer some industries over others), it was not appropriate to calculate Cronbach’s alpha. The two categories of items were averaged separately, with higher scores indicating a greater willingness to work in a particular category of industry.

ACI = Accommodation and Catering Industry; MAI = Manufacturing Industry; TWPS = Transportation, Warehousing, and Postal Services; AFAHF = Agriculture, Forestry, Animal Husbandry, and Fishery; RI = Retail Industry; MI = Mining Industry; RSLBS = Residential, Leasing, and Business Services; CI = Construction Industry; EHGWPSI = Electricity, Heat, Gas, and Water Production and Supply Industry; WI = Wholesale Industry; FII = Financial and Insurance Industry; CSEI = Culture, Sports, and Entertainment Industry; REI = Real Estate Industry; PA = Public Administration; MSW = Medical and Social Work; ITSITS = Information Transmission, Software, and Information Technology Services; SRTS = Scientific Research and Technical Services; WCEPFM = Water Conservancy, Environment, and Public Facilities Management; EI = Education Industry.

### 3.4. Statistical Analysis

**Figure 3:**
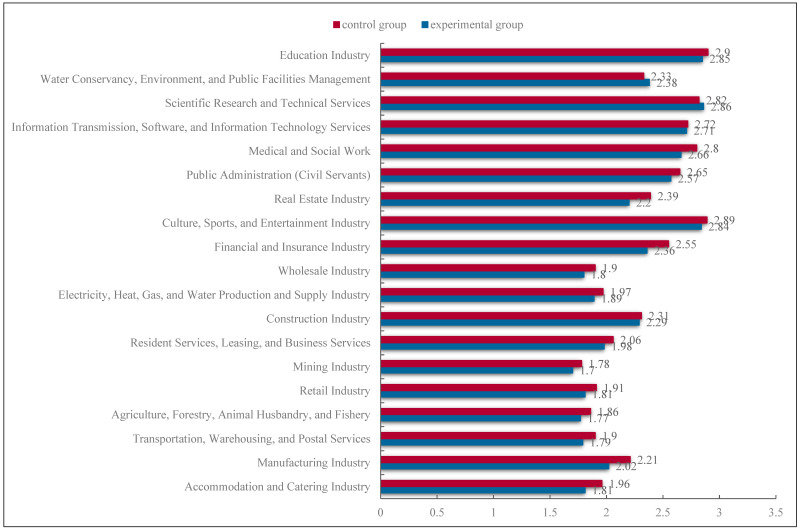
Willingness scores of adolescents to work in 19 different industries, from top to bottom, the proportion of each job being replaced by AI increases.

The statistical analysis is divided into three parts. First, the age and gender of the experimental and control groups were tested. An independent samples *t*-test was used to test whether there was a significant difference between the ages of the experimental and control groups. The results showed that there was no significant difference between the age of the experimental group (*M* = 13.89) and the age of the control group (*M* = 13.90). A cross-tabulation of experimental and control groups and gender was carried out and a chi-square test was performed, indicating that there was no difference between them (*χ*^2^(1) = 0.18, *p* = 0.67 > 0.05). We then carried out descriptive statistics (means and standard deviations) to show the basic situation of the variable; see Table 2. The career preferences of the control group and the experimental group are shown in Figure 3. We then used independent samples *t*-tests to examine the preferences for high- and low-replaceable jobs between the experimental and control groups. Subsequently, we conducted a simple moderation effect analysis using the process macro Model1 in SPSS 26.0 (Hayes, 2013) to analyze the main effect of the independent variable.

## 4. Results

Table 2 shows the means and standard deviations of all independent variables.

**Figure 4:**
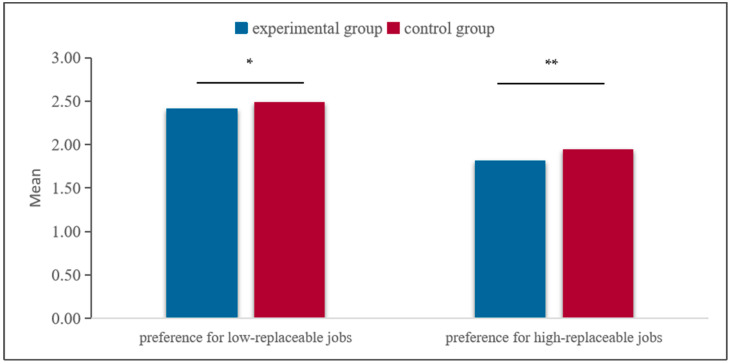
Differences test of the experimental group and the control group. Note: ** *p* < 0.01, * *p* < 0.05.

Firstly, an independent samples *t*-test was conducted to examine the differences in preferences for high-replaceable jobs between the experimental group and the control group. The results indicated a significant main effect of the experimental treatment, *t*(1831) = −2.92, *p* = 0.004 < 0.01, Cohen’s d = 0.60. Participants in the control group (*M* = 1.94) showed a significantly greater preference for high-replaceable jobs compared to those in the experimental group (*M* = 1.81). Additionally, an independent samples *t*-test was conducted to examine the differences in preferences for low-replaceable jobs. The results also revealed a significant main effect of the experimental treatment, *t*(1831) = −2.00, *p* = 0.045 < 0.05, Cohen’s d = 0.52. Participants in the control group (*M* = 2.48) showed a significantly greater preference for low-replaceable jobs compared to those in the experimental group (*M* = 2.41). The results are shown in Figure 4.

A simple moderation analysis was conducted using SPSS and the PROCESS macro (Hayes, 2013) to examine whether the relationship between independent variables (familiarity with AI, trust in AI, and positive attitude towards AI) and adolescents’ preference for low-replaceable jobs was moderated by the experimental conditions. The dependent variable was the preference for low-replaceable jobs, while the moderator was the experimental condition (information about job replacement by AI vs. control). Gender and age were included as covariates. In the PROCESS analysis, we specified Model 1 to test the interaction effects between each independent variable and the experimental condition. The results, as shown in Table 3, indicated no significant interaction effects for any of the independent variables. However, the main effects of familiarity with AI, trust in AI, and positive attitude towards AI were significant and negatively associated with adolescents’ preference for low-replaceable jobs.

## 5. Discussion

The present study examines the effects of adolescents’ prior attitudes and immediate knowledge of AI on career preference. Overall, our study found that immediate knowledge significantly and negatively predicted adolescents’ preference for high- and low-replaceable jobs. In terms of prior perception of AI and attitudes towards AI, familiarity with AI, trust in AI, and positive attitude towards AI will make adolescents more likely to choose low-replaceable jobs.

### 5.1. The Influences of Prior Perception of AI and Attitude Towards AI on Career Preference

Familiarity with AI influenced adolescents’ career preference, which is consistent with our H1, and it positively predicted adolescents’ preference for low-replaceable jobs. The greater the transparency of an AI system, the greater the participants’ trust and perceived usability of the system (Schaefer et al., 2016). In one study, the majority of participants having used AI expressed the belief that robots cannot replace the human touch, interaction, and emotional support that we need (Wolbring & Yumakulov, 2014). We infer that people who are more familiar with AI have a better understanding of which jobs can be replaced by AI, and they are more likely to choose jobs that require collaboration with AI to perform certain tasks and perform tasks that cannot be replaced by AI.

Trust in AI influenced adolescents’ career preference, which is consistent with our H1, and it positively predicted adolescents’ preference for low-replaceable jobs. Lack of trust is one of the main barriers preventing people from taking full advantage of the benefits offered by AI (Gillath et al., 2021). Working with robots in the workplace can help employees improve their skills development and job satisfaction, thereby reducing their burnout (Smids et al., 2020). Robots in the workplace can provide workers with more self-regulatory resources, reduce complex and boring situations and occupational accidents and injuries, and achieve the most positive and beneficial integration of robots and humans (Fernandez et al., 2012). We can infer that adolescents with higher levels of trust in AI tend to choose jobs that achieve long-term beneficial integration with AI (such as low-replaceable jobs), maximizing the value of both.

Positive attitude towards AI influenced adolescents’ career preference, which is consistent with our H1, and it positively predicted adolescents’ preference for low-replaceable jobs. AI is developing its capabilities at an increasing rate, but it is also creating opportunities at an increasing rate, and the fact that exposure to robots makes people feel anxious about job insecurity may be largely due to subjective assessments (Yam et al., 2022). Therefore, individuals with a positive attitude towards AI are more likely to see and embrace the opportunities AI brings, rather than only perceiving potential threats, so they prefer to let AI handle high-replaceable tasks while they choose jobs that require more skills and creativity. While negative attitude is different, there is a significant correlation between negative attitudes towards AI and the fear of job loss (Persson et al., 2021). One study explored the impact of negative attitudes on human–robot interaction and found that negative attitudes can lead to less trust in robots, thus affecting the effectiveness of human–robot interaction (Nomura et al., 2006). We can infer that due to the negative attitude primarily affecting their acceptance of AI and their fear of job loss, these emotional reactions lead people to focus more on short-term job security when choosing a career, rather than on the replaceability of the job itself. Therefore, their career choices may be more diverse and not limited to high or low-replaceable jobs. On the other hand, this study only considers replaceability as the indicator of career preference, which may not be comprehensive enough to conclude that negative attitudes influence career preferences.

### 5.2. The Influence of Immediate Knowledge of AI on Career Preference

The materials on the AI job replacement crisis reduced adolescents’ willingness to choose both high- and low-replaceable jobs. After reading these materials, adolescents might have an increasing overall awareness of the AI replacement crisis, leading to a sense of uncertainty and job insecurity which is defined as the perceived powerlessness to maintain desired continuity in a threatened job situation (Greenhalgh & Rosenblatt, 1984), and this uncertainty and insecurity make them suspect both low- and high-replaceable jobs. Research suggests that how individuals perceive AI significantly influences their career-related decisions. For instance, if AI is perceived as an assistant rather than a threat, individuals are more willing to accept it and are likely to avoid careers that are highly replaceable by AI (Hengstler et al., 2016). Similarly, a study in high-tech firms found that while AI usage can enhance employees’ innovative behaviors, the perception of high job replaceability by AI suppresses this effect (Verma & Singh, 2022). This implies that perceptions about AI not only affect current workplace behaviors but also influence long-term career planning, such as choosing roles that are less vulnerable to automation. Therefore, equipping adolescents with comprehensive AI knowledge can help them critically assess career opportunities and make informed decisions in an AI-driven environment. As in the classification of career decision difficulties (Gati et al., 1996), insufficient information is a common difficulty in the career decision-making process. The absence of career assessments and decision-making anxiety can lead to difficulties in career-related decisions (Priyashantha et al., 2023). So we can infer that after receiving instant knowledge related to AI, adolescents may feel they lack sufficient understanding of AI or encounter inconsistent information, leading to greater decision-making difficulties, thereby reducing their preference for both low-replaceable and high-replaceable jobs.

### 5.3. Implications, Future Directions, and Limitations

The present study has several theoretical contributions. First, this study integrated and experimentally examined the roles of prior perception, attitude, and immediate knowledge of AI in affecting adolescents’ career preference within a unified framework. Existing studies have explored adolescents’ concerns about the impact of AI on careers, but they have not specifically examined current attitudes towards AI or how different attitudes affect career choices. Second, the measures of the dependent variables (high- and low-replaceable career choices) collected in this study are more comprehensive and objective, and provide referenceable measures for subsequent research, and it is an approach that can better measure adolescents’ career preference.

The present study has important practical implications: first, the research findings suggest that we should take measures to enhance students’ familiarity with AI and trust in AI and cultivate their positive attitudes towards AI. This will help them be more likely to choose low-replaceable jobs in their future career choices, thereby improving employment sustainability. Second, our study shows that experimental immediate manipulation knowledge can influence adolescents’ career choices to some extent. This tells us that in the era of AI, it is important to provide enough AI-related information to teenagers to help them make career decisions, especially for those who have limited exposure to AI or limited knowledge of AI, as providing AI knowledge can prevent them from being marginalized due to information asymmetries. It also helps them better assess job stability and career prospects, enabling them to adapt to changes in the future job market. In addition, career counseling can help adolescents acquire more information about AI and future careers and assist them in coping with the psychological conflicts caused by inconsistent information.

The findings of this study should also be considered within the broader educational context. In many regions, educational systems are increasingly emphasizing the integration of digital literacy and future skills into curricula to prepare students for a rapidly evolving workforce. However, varying levels of resources and infrastructure can lead to disparities in how effectively schools address topics related to AI. Therefore, our results highlight a pressing need for educational policies that support equitable access to AI literacy programs. For policymakers, we recommend: (1) investing in teacher professional development focused on AI and its social impacts; (2) promoting the creation of age-appropriate learning resources that explain AI technologies for students; and (3) fostering collaborations between educational institutions and the technology industry to ensure that educational content remains relevant and forward-looking. Such initiatives could empower adolescents to make more informed career choices in the AI-driven era.

However, our study has some limitations. Firstly, this study is based on the background of AI education and culture in China. However, according to the Artificial Intelligence Index Report 2024, there are significant differences among countries in terms of the use of AI, attitudes towards AI, and the level of AI education (Perrault & Clark, 2024), and career choices vary in different economic environments and educational systems, thus affecting the generalization. Secondly, this study used a self-report method, which is a single method and may not reflect the true situation of the participants due to social desirability. Further research can be conducted in the future using more diverse methods. Thirdly, this study used the questionnaire method to conduct the experiment, but there was no material presented for the control group. There is a certain lack of rigor in the experimental manipulation, and future studies can be improved on this basis. Finally, career choice is a complex process influenced by numerous internal and external factors, including parents, school, and the environment (Steiner et al., 2019), even based on the environment and opportunities. In our study, we only explored adolescents’ career preferences, which may be temporary. Future research could incorporate more factors to explore this. Furthermore, while our sample was drawn from a specific grade, future studies could intentionally examine differences across wider age or developmental spans (e.g., comparing early, middle, and late adolescents) to understand how the relationships observed here might evolve.

## 6. Conclusions

The purpose of this study was to explore whether prior perception of AI, attitudes towards AI and immediate knowledge of AI influence adolescents’ preference for low- and high-replaceable jobs. The AI job replacement crisis will make adolescents less likely to choose low or high-replaceable jobs, indicating the importance of offering adolescents AI knowledge. Familiarity with AI, trust in AI, and positive attitude towards AI will make adolescents more likely to choose low-replaceable jobs.

## Figures and Tables

**Table 1:** Difference test of included and excluded participants.

Variables	Included Participants (*n* = 836)	Excluded Participants (*n* = 43)	*χ* ^2^
Gender			2.71
male	437	28	
female	399	15	
Residence			
City	797	41	0.96
Town	31	1	
Countryside	8	1	
Only child			1.48
yes	684	32	
no	152	11	

Note: Chi-square test *p*-values for the comparisons are as follows: Gender: *p* = 0.10; Residence: *p* = 0.62; Only child: *p* = 0.22.

**Table 2:** Descriptive statistics.

	Experimental Group		Control Group	
Variable	*M*	*SD*	*M*	*SD*
Familiarity	2.22	0.67	2.32	0.69
Trust	2.71	0.56	2.70	0.52
Positive attitude	3.11	0.50	3.10	0.46
Negative attitude	2.43	0.56	2.38	0.57

Note: *M*, means; *SD*, standard deviations.

**Table 3:** The main effect of the independent variable in the simple moderation effect analysis.

Variable	Low-Replaceable Job			High-Replaceable Job		
	β	*t*	*p*	β	*t*	*p*
Familiarity	0.15	4.30	<0.001	0.03	0.94	0.35
Positive attitude	0.12	3.45	<0.001	−0.03	−0.70	0.49
Negative attitude	0.03	0.95	0.34	0.02	0.51	0.61
Trust	0.11	3.12	0.002	0.01	0.28	0.78

Note: Multiple linear regression analysis.

## Data Availability

The data that support the findings of this study are available from the corresponding author upon reasonable academic request. All questionnaires and materials used in the study can be found in the Supplementary Materials.

## Supplementary Materials

The following supporting information can be downloaded at: https://www.mdpi.com/article/10.3390/bs16010072/s1, Questionnaires S1: Version A and Version B.

## Abbreviations

The following abbreviations are used in this manuscript:

CFI	Comparative Fit Index
TLI	Tucker–Lewis Index
RMSEA	Root Mean Square Error of Approximation
AI	Artificial Intelligence
STF	Systems Theory Framework
IRB	Institutional Review Board
SSRIT	Social Service Robot Interaction Trust
CFA	Confirmatory Factor Analysis
ICILS	International Computer and Information Literacy Study
ICT	Information and Communication Technology
STARA	Smart Technology, Artificial Intelligence, Robotics, and Algorithms
HRI	ACM/IEEE International Conference on Human–Robot Interaction

## Appendix A

The following are the materials presented to the experimental group (translated version):

Please read the following material from an internationally renowned research institute. After reading, please make a selection.

An internationally renowned research institute has conducted a study on the likelihood of job positions across various industries being replaced by artificial intelligence in the future. The research findings are as follows:
