# Supplementary material for: The Influence of Prior Perception, Attitude, and Immediate Knowledge of AI on Adolescents’ Preferences for High- and Low-Replaceable Jobs
Source: Behav Sci (Basel). 2026 Jan 5;16(1):72. doi: 10.3390/bs16010072 (PMC12837924; doi:10.3390/bs16010072)
Supplement: Supplementary file 1 [file behavsci-16-00072-s001.zip › Questionnaires S1/Version A.pdf]

Artificial intelligence (AI) refers to the simulation of human intelligence processes by machines, especially computer systems. This includes learning (the automated acquisition and use of information), reasoning (drawing approximate or definitive conclusions based on rules), and self-correction. Applications of AI span fields such as expert systems, speech recognition, and machine vision. Examples include personal assistants (like Siri, Xiao AI, Xiao Du, Tmall Genie, etc.), medical diagnostic aids, autonomous vehicles, and smart robots, among others. Below are some perspectives on AI. Please mark "" on the number corresponding to the option that best reflects your actual views.

1. The following statements describe familiarity with AI. Do you agree with the following statements?

	Strongly Disagree	Disagree	Agree	Strongly Agree
1. I know a lot about AI.	①	②	③	④
2. I am very familiar with AI.	①	②	③	④
3. I possess a lot of knowledge about AI-related services.	①	②	③	④
4. Compared to others, I am more familiar with AI.	①	②	③	④

2. The following are descriptions regarding AI. Do you agree with the following statements?

	Strongly Disagree	Disagree	Agree	Strongly Agree
1. AI progress generally improves living conditions.	①	②	③	④
2. AI helps us better understand the world.	①	②	③	④
3. AI is valuable to society.	①	②	③	④
4. AI progress brings many social benefits.	①	②	③	④
5. Using AI makes people more isolated in society.	①	②	③	④
6. Jobs will decrease as AI develops.	①	②	③	④
7. AI will destroy humanity.	①	②	③	④
8. Using AI may harm people's health.	①	②	③	④

3. Think about your past AI use.Do you agree with the following statements?

	Strongly Disagree	Disagree	Agree	Strongly Agree
1. The AI I used performed well.	①	②	③	④
2. The AI I used performed excellently.	①	②	③	④
3. The AI I used performed stably.	①	②	③	④

	Strongly Disagree	Disagree	Agree	Strongly Agree
4. The AI I used was very useful.	①	②	③	④
5. I trust that the AI I used acted in my best interest.	①	②	③	④
6. The AI I used did its best to help when I asked.	①	②	③	④
7. The AI I used cared about my well-being.	①	②	③	④
8. The AI I used was honest with me.	①	②	③	④
9. I believe the AI I used was honest.	①	②	③	④
10. The AI I used was sincere.	①	②	③	④
11. I feel nervous using AI in front of others.	①	②	③	④
12. I feel very nervous when facing AI.	①	②	③	④
13. I am afraid of AI.	①	②	③	④
14. I do not trust AI.	①	②	③	④

Please read the following material from an internationally renowned research in-stitute. After reading, please make a selection.An internationally renowned research institute has conducted a study on the like-lihood of job positions across various industries being replaced by artificial intelligence in the future. The research findings are as follows:

Industry classification	Likelihood of being replaced by AI
Accommodation and Catering Industry	73%
Manufacturing Industry	60%
Transportation, Warehousing, and Postal Services	60%
Agriculture, Forestry, Animal Husbandry, and Fishery	57%
Retail Industry	53%
Mining Industry	51%
Residential, Leasing, and Business Services	49%
Construction Industry	47%
Electricity, Heat, Gas, and Water Production and Supply Industry	44%
Wholesale Industry	44%
Financial and Insurance Industry	43%
Culture, Sports, and Entertainment Industry	41%
Real Estate Industry	40%
Public Administration	39%
Medical and Social Work	36%
Information Transmission, Software, and Information Technology Services	36%
Scientific Research and Technical Services	35%
Water Conservancy, Environment, and Public Facilities Management	35%
Education Industry	27%

How willing do you think you would be to work in the following industries when you are about 30 years old?

	Strongly Disagree	Disagree	Agree	Strongly Agree
1. Accommodation and Catering Industry	①	②	③	④
2. Manufacturing Industry	①	②	③	④
3. Transportation, Warehousing, and Postal Services	①	②	③	④
4. Agriculture, Forestry, Animal Husbandry, and Fishery	①	②	③	④
5. Retail Industry	①	②	③	④
6. Mining Industry	①	②	③	④
7. Resident,Leasing,and Business Services	①	②	③	④
8. Construction Industry	①	②	③	④
9. Electricity, Heat, Gas, and Water Production and Supply Industry	①	②	③	④

	Strongly Disagree	Disagree	Agree	Strongly Agree
10. Wholesale Industry	①	②	③	④
11. Financial and Insurance Industry	①	②	③	④
12. Culture, Sports, and Entertainment Industry	①	②	③	④
13. Real Estate Industry	①	②	③	④
14. Public Administration	①	②	③	④
15. Medical and Social Work	①	②	③	④
16. Information Transmission, Software, and Information Technology Services	①	②	③	④
17. Scientific Research and Technical Services	①	②	③	④
18. Water Conservancy, Environment, and Public Facilities Management	①	②	③	④
19. Education Industry	①	②	③	④

The questionnaire ends here. Please check carefully to see if any questions have been left unanswered. Thank you for your support!
